# Do Chinese Teachers Perform Emotional Labor Equally? Multi-Group Comparisons Across Genders, Grade Levels and Regions

**DOI:** 10.3389/fpsyg.2019.00190

**Published:** 2019-02-07

**Authors:** Shenghua Huang, Hongbiao Yin, Jiwei Han

**Affiliations:** ^1^Faculty of Education, The Chinese University of Hong Kong, Shatin, Hong Kong; ^2^School of Mathematics and Statistics, Northeast Normal University, Changchun, China

**Keywords:** teacher emotion, emotional labor, teaching satisfaction, measurement invariance, China

## Abstract

The emotional aspects of teaching are important and teachers’ emotional labor, or, how teachers manage emotions at school, has been attracting more and more attention recently. Using multi-group structural equation modeling, this study investigated the measurement invariance of, and the relationships between, teachers’ emotional labor strategies and teaching satisfaction. Participants included teachers from primary and secondary schools in Hong Kong and mainland China. Three sets of group comparisons have been made between female and male teachers, between primary and secondary school teachers, and between teachers in Hong Kong and mainland China. The multi-group invariance tests showed no significant subgroup differences in the measurement and structural models. Thus, there was no difference of ‘kind.’ However, some differences of ‘degree’ were observed across genders, grade levels and regions. These differences in the relationship between surface/deep acting and teaching satisfaction can be attributed to the possible influence of some cognitive factors and socio-cultural contexts. With due methodological rigor, the results of this study provide deeper understanding of teachers’ emotional labor and its relationship with teaching satisfaction.

## Introduction

Over the past decades, researchers have recognized and investigated the roles of emotions and emotion regulation in the workplace (Hochschild, 1983; Grandey, 2000; Grandey and Melloy, 2017). Particularly, emotional labor has been conceptualized as the process during which employees manage their emotions for a wage (Hochschild, 1983). Researchers have suggested that emotion regulation is at the core of emotional labor, and that individuals may use different emotional labor strategies (ELSs) to regulate their emotions at work (Grandey, 2000; Humphrey et al., 2015). There are three types of ELSs: surface acting, deep acting (DA) and the expression of naturally felt emotions. Surface acting (SA) is related to hiding or faking emotions and thus to a dissonance between expressed emotions and inner feelings. Deep acting (DA) refers to modifying or changing one’s inner feelings at the outset and naturally expressing the required emotions (Hochschild, 1983). The expression of naturally felt emotions (ENFE) means expressing felt emotions without any attempts to modify or fake them (Diefendorff et al., 2005). Researchers also suggested that individuals can be classified into different profiles according to their different levels of trying (i.e., DA) and faking (i.e., SA) (Gabriel et al., 2015). There is a consensus that organizational contexts such as emotional demands of the job could be seen as the situational cues triggering the emotional labor process, while organizational outcomes such as performance and turnover rate, and employee’s psychological outcomes, such as job satisfaction and burnout, could be seen as the results of emotional labor (Grandey, 2000; Grandey and Melloy, 2017). In general, SA is stressful and resource-depleting, and thus is negatively related to employee health and well-being. DA, as a strategy adopted with goodwill, is both stressful and rewarding, while ENFE seems to be always adaptive (Hülsheger and Schewe, 2011; Humphrey et al., 2015).

Teacher emotion is important for not only the motivational and emotional functioning of students, but also the professional and affective well-being of teachers (O’Connor, 2008; Jennings and Greenberg, 2009; Cheung et al., 2011). In the field of educational research, emotional labor of teachers have attracted more and more attentions. Several studies have investigated the relationships between teachers’ emotional labor and their well-being (Zhang and Zhu, 2008; Cheung et al., 2011; Yin, 2012; Yin et al., 2016, 2017; Sarraf et al., 2017). The results were quiet similar to those obtained in non-educational setting, except that the results of teachers sometimes depicted some bright sides of emotional labor (Humphrey et al., 2015; Yin et al., 2017).

Another group of researchers, on the other hand, have explored how individuals differ in the ways that they express and regulate their emotions and in their consequential well-being outcomes (Maxwell et al., 2005; De Vaus et al., 2018; Mastracci and Adams, 2018). In conducting these measurement-based across-group comparisons, researchers have consciously or unconsciously made assumptions that different groups of participants perceive and respond to the measurements equally or that the measurements are invariant across groups (Vandenberg and Lance, 2000). Nevertheless, most studies have not explicitly tested measurement invariance or at least partial invariance before conducting substantive comparisons across groups. Thus, it is difficult to give sound interpretations of the results as the significant differences detected could be due to either measurement variance or ‘true’ differences between groups (Vandenberg and Lance, 2000).

When it comes to the comparisons of the gender, grade levels and cultural differences in the emotional labor or well-being of teachers (e.g., Hargreaves, 2000; Oshagbemi, 2000; Marston et al., 2005; Antoniou et al., 2006), similar methodological weakness exists. It is important to go beyond testing mean differences in the teachers’ use of ELSs or teaching satisfaction among different subgroups, and explore whether and how these categorical factors may moderate the relationships between teachers’ use of ELSs and their well-being. In addition, researchers interested in the universal aspects of emotion and cultural differences in emotion have often turned to China because of its cultural specificities (Yik, 2010). The results of cross-cultural studies have shown significant differences in the perception and regulation of emotions between Chinese and Western cultures (Maxwell et al., 2005; Allen et al., 2014; De Vaus et al., 2018; Mastracci and Adams, 2018). However, with some exceptions (e.g., Wong et al., 2007; Fung and You, 2011), few studies have looked at the differences in the experience and regulation of emotions within China which is famous for its regional diversity.

Considering these gaps in literature and the regional diversity of China, this study aims to use multi-group structural equation modeling (SEM) and compare the gender, grade levels and cultural differences in the emotional labor and well-being outcome within the educational context. Drawing on two data sets from Hong Kong and Mainland China, this study tries to answer the following two questions: (1) Are the measurements of teacher ELSs and teaching satisfaction invariant across gender, grade levels and regions? (2) And are the paths between ELSs and teaching satisfaction invariant across gender, grade levels and regions?

This study contributes to the literature in the following two aspects. First, we compare individual differences in terms of teachers’ emotional labor and its relationship with teaching satisfaction. While a great number of studies have investigated emotional labor and synthesized previous results, few studies have examined and compared the individual or cultural differences in emotional labor and how these categorical factors may moderate the relationship between emotional labor and its outcomes. Second, we better address the methodological rigor than previous studies by using multi-group SEM to confirm measurement invariance before conducting substantive comparisons across groups. These procedures help to identify the differences between groups. In the following sections, we first review previous theoretical and empirical results and make hypotheses accordingly. We then introduce the data and method used in the analysis. Finally, we report and discuss our results. Limitations are also discussed.

## Theoretical Background and Literature Review

### Emotional Labor and Teaching Satisfaction

Emotional labor is a common job requirement faced by employees in service sector. Hochschild (1983) first adopted the term ‘emotional labor’ to describe the process where service employees create certain facial expressions and use body or verbal language when interacting with the customers (Hochschild, 1983). In order to fulfill the professional norms, teachers have to display desirable facial expressions and use proper body and verbal language in their interactions with students, parents and colleagues (O’Connor, 2008; Cheung et al., 2011; Yin et al., 2017). In other words, teachers have to perform emotional labor during school.

Job satisfaction is a common well-being indicator used in organizational studies. While well-being generally denotes how and why individuals experience their life in positive ways (DeNeve and Cooper, 1998), job satisfaction usually refers to ‘the pleasurable emotional state resulting from the appraisal of one’s job as achieving or facilitating one’s job values’ (Locke, 1969, p. 316). In educational settings, teachers’ job satisfaction, or teaching satisfaction, is defined by Ho and Au (2006) as ‘a function of the perceived relation between what one wants from one’s job and what one perceives teaching as offering or entailing’ (p. 172).

A meta-analytic review carried out by Hülsheger and Schewe (2011) showed that there were positive relationships between SA and emotional exhaustion (ρ = 0.44) and depersonalization (ρ = 0.48) while negative ones between SA and personal accomplishment (ρ = -0.10) and job satisfaction (ρ = -0.33). DA was also found to be positively related to emotional exhaustion (ρ = 0.09) but meanwhile positively related to personal accomplishment (ρ = 0.27). The results found among teachers were generally consistent with those found among a wide range of occupations. When focused on teachers’ emotional labor, empirical studies have generally suggested that various ELSs relate differently to teachers’ job satisfaction (Zhang and Zhu, 2008; Yin, 2012; Sarraf et al., 2017). For example, Sarraf et al. (2017) used a second-order structure of emotional labor, and found significant and negative relationships between emotional labor and both teaching satisfaction and performance. Zhang and Zhu (2008) found some deleterious effects of surface acting while positive effects of DA and authenticity on teacher burnout and satisfaction. Yin (2012), however, found no significant relationships between SA and both intrinsic and extrinsic job satisfaction but positive ones between job satisfaction dimensions and both DA and ENFE. A meta-analytic review of teachers’ emotional labor showed that SA was significantly related to personal accomplishment (ρ = -0.11), and DA was not significantly related to emotional exhaustion (ρ = -0.02) or depersonalization (ρ = -0.04) (Yin et al., unpublished). ENFE was found to be negatively related to emotional exhaustion (ρ = -0.26) and depersonalization (ρ = -0.47) while positively related to personal accomplishment (ρ = 0.40) and teaching satisfaction (ρ = 0.34) (Yin et al., unpublished).

In sum, SA is consistently found to be a maladaptive way, while ENFE an adaptive way to regulate emotion and that DA is positively related to individuals’ professional well-being (sense of efficacy or accomplishment) and is weakly and sometimes non-significantly related to their affective well-being (e.g., emotional exhaustion, depersonalization). Nevertheless, researchers have taken different theoretical perspectives to explain the effects of different ELSs. Hülsheger and Schewe (2011) noted three typical types of mechanisms used to explain the relationships between ELSs and individual well-being.

First, the most widely examined theories are those related to resource depletion and replenishment. Hobfoll’s (1989) conservation of resources theory suggests that each individual owns a reservoir of limited resources and that ‘people strive to retain, protect, and build resources and what is threatening to them is the potential or actual loss of these valued resources.’ Thus, individuals find the extensive depletion of resources without sufficient replenishment stressful and this can be predictive of their strain and ill-being (Humphrey et al., 2015). As such, while ENFE is related to unconscious effort or no extensive resource depletion, surface and DA are related to conscious emotion regulation and effortful self-control, and thus deplete individuals’ mental resources. Resource depletion only exists at the very early stages of DA, for example, when people try to cognitively reappraise situations. However, sustained resource depletion is related to SA as people continuously devote effort and deplete resources to hide or fake expressions throughout the whole personal interactions (Grandey, 2000; Hülsheger and Schewe, 2011). Furthermore, DA is generally characterized by sincere and positive expressions and there is thus the potential for resource replenishment due to positive feedback from the people (e.g., students or customers) with whom they interact (Yin et al., 2017). In contrast, surface actors may run the risk that any faked or insincere, although friendly, expressions could be detected by the other side, making constructive interactions difficult and leading to one’s further attempts to regulate emotions and new rounds of resource depletion (Johnson and Spector, 2007; Yin et al., 2017).

Second, theories that focus on the effects of positive and negative emotions are also helpful to explain the relationships between ELSs and individual well-being. While the preponderance of positive over negative affect *per se* is an important dimension of subjective well-being, the broaden-and-build theory highlights that positive emotions in the present also predict positive emotions in future, resulting in upward spirals of emotion and better well-being (Fredrickson, 2001). With few exceptions (e.g., debt collectors), teachers and most other service employees are required to express more positive than negative emotions during work, although teachers also have the autonomy to express negative emotions when necessary (Hochschild, 1983; Yin, 2016). Naturally felt positive emotions and those achieved through DA are beneficial and contribute to teachers’ job satisfaction and long-term well-being. In contrast, faked smiles (SA) may not trigger upward spirals, but rather give teachers a sense of inauthenticity, which is also the focus of the third line of theories (Yin et al., 2017; Grandey and Melloy, 2017).

The third line of theories proposes that people have the desire to authentically express themselves. As a result, SA, characterized by the discrepancy between inner feeling and external expression (or emotional dissonance), is considered to be extremely harmful to individuals’ well-being (Grandey, 2000; Johnson and Spector, 2007; Humphrey et al., 2015; Grandey and Melloy, 2017). This tendency of emotional consistency is largely rooted in theories related to self-concept and self-consistency. Researchers have suggested that by interacting with the external world, individuals build their self-concept about who they are and are not (personal identity) and how they are connected with and distinguished from social groups (social identity) (Shamir et al., 1993). Individuals are motivated to maintain self-consistency, which refers to the compatibility of different self-concept components, the continuity of self-concept overtime, and the correspondence between self-concept and behaviors (Shamir et al., 1993). Thus, the consistency between felt emotions and expressed emotions is especially important, given the assumption that people believe that they should be authentic and honest (Hülsheger and Schewe, 2011; Humphrey et al., 2015).

Thus, based on previous theoretical and empirical results, we generally hypothesized the relationships between ELSs and teaching satisfaction as following:

H1a: SA is negatively and significantly related to teaching satisfaction.
H1b: DA is positively although weakly related to teaching satisfaction.
H1c: ENFE is positively and significantly related to teaching satisfaction.

### Sources of Individual Differences

Emotions are thought to be universally understandable. While there are universally agreed common practices to express emotions and feelings, considerable effort has been devoted to examining the individual differences with regard to the valances, frequencies and intensities of expressed emotions (Fabrizio et al., 2015). For example, people in Western cultures tend to express their emotions more directly and with higher intensity. In contrast, the Chinese culture advocates reservation and the golden mean, where strong emotions, no matter whether positive or negative, are considered to be disruptive and the expressions of them should be controlled (Fabrizio et al., 2015; Mastracci and Adams, 2018). Women are thought to be more vulnerable to negative emotions, such as anxiety and depression, than men (Chan and Lee, 2006; Johnson and Spector, 2007). Additionally, individuals differ in the ways that they encode, decode and regulate their emotions. For example, individuals may find it difficult to correctly ‘read’ the facial expressions of people from unfamiliar cultures (Wong et al., 2007). While females are thought to be more emotionally expressive, they also tend to use maladaptive ways to regulate their emotions (Grandey, 2000; Maxwell et al., 2005; Johnson and Spector, 2007).

Limited effort has been devoted to examine the individual differences in the mechanisms explaining the relationships between ELSs and individual well-being. For example, the relative importance given to authenticity varies across cultures. Studies have also suggested that women value authenticity more and use less deception than men (Hogue et al., 2013). Thus, SA and feelings of being inauthentic may be related to the well-being of men and women differently. Furthermore, teachers who attach more importance to lecture delivery and classroom management (or technical competence) may allocate their resources differently compared to those who admit the importance of students’ motivational and emotional functioning (or human interaction and emotional understanding) (O’Connor, 2008). For instance, the latter may assign more resources to emotion regulation tasks, perceive these efforts as worthwhile, and being less vulnerable to emotional labor and resource depletion. Similarly, Corcoran and Tormey (2013) suggested that when the cognitive load is high, for example, when student teachers have to learn and use different teaching pedagogies and skills, the predictive effects of emotional intelligence (emotional competence) on student teachers’ increased performance may fade due to cognitive overload.

Nevertheless, studies have also provided some preliminary evidence for the differences in genders, grade levels and regions in terms of individuals’ emotion regulation and well-being, though some of these studies were conducted using qualitative methods and few quantitative studies had based their substantive hypothesis tests on the prerequisite of measurement invariance.

#### Gender Differences

A relatively high portion of female employees is an important characteristic of occupations that demand intensive emotional labor (Hochschild, 1983). Women are generally thought to be better at coping with other people and attribute more importance to friendly and harmonious social relationships compared to men (Grandey, 2000; Chan and Lee, 2006). According to the literature, in a relatively low social status and position, women (and other minorities) are more likely to suffer from emotional attacks from others. They are also more likely to hide their negative emotions (e.g., anger and aggressiveness) and try their best to be as friendly and humble as possible (Hargreaves, 2000; Erickson and Ritter, 2001). The literature has further suggested that while women are more motivated than men to manage their emotions during work, they also tend to use maladaptive emotional labor strategies (e.g., suppressing and faking their inner feelings), which may lead to more symptoms of stress in the long term (Chan and Lee, 2006; Johnson and Spector, 2007; Grandey and Melloy, 2017). Similarly, researchers have found that, in attempts to resolve negative emotions, women are more likely to think unpleasant events and feelings (e.g., anger) over and over again (i.e., rumination), while men are more likely to use distraction or attention deployment; rumination is unfortunately positively related to further negative emotions while distraction is not related or negatively related to them (Maxwell et al., 2005). However, mixed findings have been reported. Johnson and Spector (2007), for instance, found that SA was more negatively related to women’s well-being. This was probably because women want to adhere to their traditional roles of caregivers and value authentic emotional expression more than men. In contrast, although Erickson and Ritter (2001) assumed that the management of agitation leads to more burnout and inauthenticity in women, they found no significant gender difference in terms of these negative effects.

Likewise, the effects of demographic characteristics on teacher burnout are not salient or consistent. While Oshagbemi (2000) found that female university teachers were slightly more satisfied with their job than their male counterparts, Antoniou et al. (2006) found higher levels of stress and burnout in female school teachers.

#### Grade Level Differences

Primary and secondary schools focus on different aspects of student development. While primary schools take more responsibility for students’ social development, secondary schools give priority to their academic results (Marston et al., 2005; Jennings and Greenberg, 2009). Further, adolescents in secondary school seem to encounter more age-related emotional problems from the outside in compared to pupils in primary school (Hargreaves, 2000). While there are limited empirical results for the grade-level differences in teachers’ emotions and emotional labor, Hargreaves (2000) found that primary school was characterized by greater emotional intensity, while secondary school teachers may perceive intensive emotions as something to be avoided. When primary school teachers integrate delight and excitement into their teaching and express anger and agitation more directly, their secondary school counterparts try to solve students’ emotional problems outside the classroom and attend to the emotional needs of individual students, rather than those of the whole class (Hargreaves, 2000). Similarly, apart from teaching, primary school teachers must devote more effort to take care of the students’ daily life and spend more time with them (Jennings and Greenberg, 2009). Furthermore, majoring in a specific subject and teaching one subject to different classes, secondary school teachers attribute more importance to the subjects they teach compared to their primary school counterparts who have more general college majors and teach several subjects to the same class (Marston et al., 2005). The lack of emotional interactions between teachers and their students during class is a problem for secondary school education as it is important for teachers to not only regulate the negative emotions of students, but also promote positive ones in their learning (Hargreaves, 2000; Jennings and Greenberg, 2009).

In terms of their well-being, Marston et al. (2005) found that primary school teachers were more satisfied with their job compared to their secondary school counterparts. Although females were more likely to feel satisfied with their jobs and the proportion of females in primary schools was larger, there was no significant gender difference in teachers’ job satisfaction, suggesting that this difference may be caused by other factors than the percentage of female teachers (Marston et al., 2005). Researchers have also suggested that after a teacher preparation program, primary student teachers felt more capable than secondary student teachers. The intimate relationships and emotional understanding between primary school student teachers and their students may contribute to their pedagogical skills in care and concern (Wong et al., 2008).

#### Regional Differences

This study concerned the regional differences between Hong Kong and mainland China. Hong Kong is a special administrative region of China. It was a British colony for about one and a half centuries before China reclaimed sovereignty over the region in 1997 (Chan and Lee, 2006). The ‘One Country, Two Systems’ policy, which guarantees the region’s autonomy to run its capitalist system and operate its own policies and laws, currencies, socio-administrative and educational systems for at least 50 years, has been applied to Hong Kong since then (Li and Bray, 2007). Given this historical background, there are both similarities and differences between Hong Kong and mainland China.

The existence of sub-cultures among Hong Kong and mainland China makes it important to compare the emotional labor of teachers from the two regions. People from Hong Kong and mainland China are believed to follow the Confucian cultural traditions and share some basic values (Chan and Lee, 2006; Wong et al., 2007). The Confucian traditions generally advocate reservation and the golden mean, self-control and social harmony, as well as paternalism and family ties (Chan and Lee, 2006; Fabrizio et al., 2015). However, some researchers have also highlighted the existence of sub-cultures within Chinese society (Fung and You, 2011). The most salient sub-cultural difference between Hong Kong and mainland China is the extent to which an individual or the society accepts the unequal distribution of power (i.e., power distance) (Farh et al., 2007). Greatly influenced by the Western modes of organizational structure and personnel design, the power distance of institutions and organizations in Hong Kong is relatively low compared to those in mainland China (Kennedy et al., 2004; Farh et al., 2007). Moreover, while people from both Hong Kong and mainland China value family ties and the emotional dependence of kin, those from mainland China are more accepting of authority and hierarchy within the family (Fung and You, 2011). Even though strong emotions should be controlled rather than expressed in Chinese culture, the elderly in mainland China are more willing and able to restore the hierarchy within the family by expressing their anger and aggression toward kin (Fung and You, 2011; Fabrizio et al., 2015). Similarly, compared with their counterparts in Hong Kong, teachers in mainland China have more authority in their interactions with students and parents. This may further influence their perceived importance of emotional labor and the strategies that they adopt.

In addition, Hong Kong is more modernized and urbanized and its average income is much higher than that of mainland China. Although the rapid economic development of mainland China is characterized by a steady growth in GDP, the society’s psychosocial development is still lagging behind Hong Kong (Kennedy et al., 2004; Chan and Lee, 2006). However, Wong et al. (2007) suggested that the effect of the economic environment on the way that individuals regulate their emotions is only minor and that there is no strong evidence to demonstrate significant cultural or value differences between people from Hong Kong and mainland China.

Although there is limited and controversial evidence in terms of individuals’ emotion regulation and well-being, some patterns can be identified. For example, although females are thought to be more emotionally expressive, they tend to use maladaptive emotional labor strategies and suppress their emotions. As compared to their primary school counterparts, secondary school teachers focus more on the cognitive rather than the affective aspects of their jobs. In addition, with higher power distance, mainland China teacher generally enjoy more authority in the workplace and thus can be more flexible in the use of ELSs. Thus, we generally made the following hypotheses:

H2: The relationships between ELSs and teaching satisfaction will vary across genders (H2a), grade levels (H2b), and regions (H2c).

## Materials and Methods

### Data and Samples

Consistent with institutional review board procedures, this study was carried out in accordance with the recommendations of Survey and Behavioral Research Ethics Committee at the Chinese University of Hong Kong with written informed consent from all subjects. All subjects gave written informed consent in accordance with the Declaration of Helsinki. The protocol was approved by the Survey and Behavioral Research Ethics Committee.

#### Mainland Sample

The sample from mainland China comprised teachers from primary and secondary schools in Beijing and Chongqing. The questionnaire survey was conducted from August 2012 through February 2013. The researchers indirectly contacted the teachers through their principals. The teachers were subsequently invited to participate in the questionnaire survey on a voluntary basis. Anonymity was guaranteed. An invitation letter was attached to the questionnaire, explaining the nature, purpose and method of the survey. It provided the participants with instructions on how to complete the questionnaire. In total, 673 primary and 608 secondary school teachers constituted the final mainland sample. There were 77.12 and 69.74% females in the primary and secondary school samples, respectively.

#### Hong Kong Sample

The Hong Kong sample comprised teachers from primary and secondary schools in Hong Kong. The questionnaire survey was conducted from November 2015 through January 2016. In total, 60 primary schools and 30 secondary schools were invited to participate in the survey. The questionnaires with an introduction letter were sealed in envelopes and sent to each school. The introduction letter clearly stated the nature, purpose and procedure of the study. The teachers were invited to participate in the survey voluntarily and anonymity was guaranteed. There were 1,115 primary and 541 secondary school teachers in the final Hong Kong sample. There were 76.41 and 58.23% females in the Hong Kong primary and secondary school samples, respectively. Twenty-four participants who did not report their gender were excluded from the analyses of gender difference.

The data collection procedures for the two samples were similar. There were 2,937 teachers in the final sample. The data analyses were based on subgroups of gender (female = 2,110, male = 803), grade levels (primary = 1,788, secondary = 1,149) and regions (mainland China = 1,281, Hong Kong = 1,656).

### Measures

#### Emotional Labor Strategies

Three ELSs were measured using an adapted version of the ELS Scale developed by Diefendorff et al. (2005). A total of 13 items were used to measure the three strategies: SA (six items; e.g., ‘I fake the emotions I show when dealing with students or their parents’), DA (four items; e.g., ‘I try to actually experience the emotions that I must show to students or their parents’), and ENFE (three items; e.g., ‘The emotions I show students or their parents match what I spontaneously feel’). For the total sample, the Cronbach’s α coefficients of SA, DA and ENFE were 0.88, 0.71, and 0.75, respectively. The ELS Scale has been used previously both within and beyond the educational setting (e.g., Gosserand and Diefendorff, 2005; Zhang and Zhu, 2008; Cheung et al., 2011; Gabriel et al., 2015). The above mentioned studies showed that the scale has good reliabilities: studies using teacher samples reported Cronbach’s α coefficients ranging from 0.78 to 0.90; studies focusing on a wide range of occupations reported Cronbach’s α coefficients ranging from 0.83 to 0.92.

#### Teaching Satisfaction

Ho and Au’s (2006) the Teaching Satisfaction Scale consists of five items and was used to measure the teachers’ job satisfaction. Example items included ‘In most ways, being a teacher is close to my ideal’ and ‘My conditions of being a teacher are excellent’. The Cronbach’s α coefficient for the total sample was 0.86. The Teaching Satisfaction Scale has been used in many previous studies and is found to have good reliabilities (e.g., Badri et al., 2013, Cronbach’s α = 0.94; Duffy and Lent, 2009, Cronbach’s α = 0.86).

### Analyses

The measurement invariance tests and equal factor means test were based on the confirmatory factor analysis (CFA). The following path comparisons were based on the SEM using Mplus 7.0. SPSS was also used to handle missing data (Expectation Maximization Algorithm) and calculate descriptive statistics.

As the absolute values of skewness were less than 3 and those of kurtosis were less than 10 (see Table 1), the normality assumption was not severely violated. The maximum likelihood estimator in Mplus was thus used for both CFA and SEM (Kline, 2005). To evaluate a single model fit, four goodness-of-fit indices were used: the Comparative Fit Index (CFI), the Tucker-Lewis Index (TLI), the Standardized Root Mean Square Residual (RMSEA), and the Standardized Root Mean Square Residual (SRMSR). The data fit is acceptable when the CFI and TLI are no less than 0.90 (the higher, the better), the RMSEA is under 0.08 (the lower, the better) and the SRMSR is under 0.10 (the lower, the better). The data fit is good when CFI and TLI are no less than 0.95 (the higher, the better), RMSEA is under 0.06 (the lower, the better), and SRMSR is under 0.08 (the lower, the better). The Chi-square statistics were reported, but not relied upon to evaluate the model fit as they are sensitive to the sample size and tend to suggest significant differences between the hypothesized model and observed data (Kline, 2005).

**Table 1:** Descriptive statistics and reliabilities for subgroups.

	Mainland (*n* = 1281)						Hong Kong (*n* = 1656)				
			
	*M*	*SD*	Skewness	Kurtosis	α		*M*	*SD*	Skewness	Kurtosis	α
SA	2.60	0.74	0.29	0.05	0.84		2.89	0.79	0.07	-0.45	0.80
DA	3.44	0.61	-0.31	1.18	0.69		3.46	0.56	-0.51	0.54	0.74
ENFE	3.66	0.65	-0.52	0.86	0.68		3.54	0.66	-0.62	0.50	0.80
TS	3.23	0.75	-0.26	0.27	0.88		3.51	0.71	-0.59	0.58	0.89

	**Primary School (*n* = 1788)**						**Secondary School (*n* = 1149)**				
			
	***M***	***SD***	**Skewness**	**Kurtosis**	**α**		***M***	***SD***	**Skewness**	**Kurtosis**	**α**

SA	2.79	0.79	0.11	-0.38	0.89		2.72	0.78	0.28	-0.14	0.87
DA	3.48	0.56	-0.42	0.57	0.70		3.41	0.61	-0.39	1.16	0.73
ENFE	3.61	0.64	-0.57	0.55	0.75		3.56	0.67	-0.58	0.78	0.75
TS	3.42	0.72	-0.49	0.43	0.88		3.35	0.77	-0.37	0.16	0.89

	**Male (*n* = 803)**						**Female (*n* = 2110)**				
			
	***M***	***SD***	**Skewness**	**Kurtosis**	**α**		***M***	**SD**	**Skewness**	**Kurtosis**	**α**

SA	2.79	0.82	0.25	-0.13	0.89		2.75	0.77	0.14	-0.41	0.88
DA	3.47	0.58	-0.39	0.83	0.72		3.44	0.58	-0.43	0.90	0.71
ENFE	3.56	0.67	-0.55	0.66	0.75		3.60	0.65	-0.59	0.67	0.75
TS	3.42	0.75	-0.45	0.49	0.89		3.37	0.73	-0.46	0.24	0.88


*SA, surface acting; DA, deep acting; ENFE, expression of naturally felt emotions; TS, teaching satisfaction.*

Following the procedures recommended by Vandenberg and Lance (2000), three stages of analyses were conducted. In the first stage, an omnibus test of the covariance invariance was conducted across the subgroups to determine if there was a significant difference between the covariance matrices of the observed items (Model 0). If there was no significant difference, no further tests of invariance were necessary. If there was a significant difference, further tests of invariance were needed to identify the sources of difference. The second stage involved testing the measurement invariance (whether observed items operationalise the latent factor invariantly across subgroups). We tested a series of models with progressively strict constraints of equal parameters across the subgroups. Model 1 (configural invariance) was used as the baseline model and required that the observed items represented the same constructs across the subgroups. Model 2 (metric invariance) was based on Model 1 and added further constraints of equal factor loadings across the subgroups. Model 3 (scalar invariance) was based on Model 2 and added further constraints of equal item intercepts across the subgroups. The third stage involved testing the substantive structural invariance of concern. We tested if the latent factor means were invariant across the subgroups (Model 4) and whether the latent factor variances, latent factor covariance and path coefficients were invariant across the subgroups (Model 5–7). Model 4 (invariant factor means) and Model 5 (invariant factor variances) were based on Model 3 and added further constraints of equal factor means and equal factor variance across the subgroups, respectively. Model 6 (invariant factor covariance) was based on Model 5, with constraints of equal factor covariance. Model 7 (invariant structural parameters) was a structural equation model based on Model 6, with the constraint of equal path coefficients across the subgroups. The recommendations of Cheung and Rensvold (2002) were used to determine if there was a significant difference between the models: a decrease in CFI greater than 0.01 (i.e., ΔCFI < -0.01) indicated that there was a significant difference between the previous and the new model. It also showed that the equality constraints placed on the new model significantly decreased the model fit of the previous model. Similarly, the results of the chi-square difference tests were reported only for reference as the results tend to suggest significant differences between the two models under comparison.

## Results

### Descriptive Statistics, Reliabilities and Correlations

Table 1 shows the descriptive statistics and reliabilities for each subgroup. The mean scores of SA (ranging from 2.60 to 2.89) were relatively lower than those of DA (ranging from 3.41 to 3.48) and ENFE (ranging from 3.54 to 3.66). This indicated the teachers’ less frequent use of SA. The skewness ranged from -0.62 to 0.29 and the kurtosis ranged from -0.45 to 1.18, indicating that the data were fairly normally distributed and could be used in further analyses. The Cronbach’s α coefficients of teaching satisfaction for each subgroup ranged from 0.88 to 0.89. The Cronbach’s α coefficients of SA, DA and ENFE for each subgroup ranged from 0.68 to 0.89, suggesting acceptable internal consistencies.

Table 2 shows the factor loadings and latent factor correlations for the subgroups, which were the CFA results of the baseline model (Model 1). The results showed that when all of the parameters were allowed to be estimated without any constraints between the subgroups, the like parameters (factor loadings and correlations) for primary and secondary school teachers were similar, as were those for male and female teachers. For example, the factor loadings of SA for primary school teachers ranged from 0.68 to 0.80. This was close to those for secondary school teachers (ranging from 0.68 to 0.85). Similarly, the correlation between SA and DA for primary school teachers was 0.43, which was close to that for secondary school teachers (*r* = 0.44). However, the differences between the like parameters for teachers from mainland China and Hong Kong were more salient. For example, the factor loadings of ENFE for teachers from mainland China ranged from 0.56 to 0.72, with a mean score of 0.64. In contrast, those for Hong Kong teachers ranged from 0.75 to 0.78, with a mean score of 0.76. The correlation between SA and ENFE for teachers from mainland China was -0.35 compared to -0.67 for teachers from Hong Kong.

**Table 2:** Factor loadings and latent factor correlations for subgroups.

	Range of loadings (mean)	1	2	3	4	Range of loadings (mean)
	***Mainland***					***Hong Kong***
	
(1) SA	0.60–0.81 (0.69)	–	0.38	-0.67	-0.34	0.73–0.83 (0.78)
(2) DA	0.53–0.65 (0.60)	0.48	–	-0.01	0.03	0.54–0.79 (0.66)
(3) ENFE	0.56–0.72 (0.64)	-0.35	0.33	–	0.41	0.75–0.78 (0.76)
(4) TS	0.70–0.88 (0.77)	-0.09	0.20	0.36	–	0.73–0.89 (0.79)
	
	***Primary School***					***Secondary School***
	
(1) SA	0.68–0.80 (0.75)	–	0.44	-0.55	-0.18	0.68–0.85 (0.73)
(2) DA	0.52–0.71 (0.61)	0.43	–	0.17	0.18	0.55–0.72 (0.64)
(3) ENFE	0.68–0.74 (0.71)	-0.56	0.09	–	0.41	0.64–0.81 (0.72)
(4) TS	0.74–0.88 (0.78)	-0.20	0.06	0.33	–	0.73–0.87 (0.78)
	
	***Male***					***Female***
	
(1) SA	0.70–0.85 (0.76)	–	0.42	-0.58	-0.18	0.68–0.80 (0.74)
(2) DA	0.55–0.69 (0.63)	0.49	–	0.10	0.11	0.52–0.73 (0.62)
(3) ENFE	0.69–0.74 (0.71)	-0.45	0.21	–	0.36	0.64–0.75 (0.71)
(4) TS	0.73–0.88 (0.79)	-0.21	0.13	0.38	–	0.74–0.87 (0.78)


*SA, surface acting; DA, deep acting; ENFE, expression of naturally felt emotions; TS, teaching satisfaction; means of factor loadings are in parentheses; correlations below the diagonal are for Mainland, primary school, and male teachers respectively; correlations above the diagonal are for Hong Kong, secondary school, and female teachers, respectively.*

### Results of the Omnibus Tests of Invariance

The first sections of Tables 3–5 show the results of the omnibus tests of the invariant covariance matrices of observed items across genders, grade levels and regions, respectively. The results in Tables 3, 4 show that the model 0 for gender comparison (χ^2^ = 253.33, *df* = 171, CFI = 1.00, TLI = 0.99, RMSEA = 0.018, SRMSR = 0.027) and the model 0 for grade level comparison (χ^2^ = 374.34, *df* = 171, CFI = 0.99, TLI = 0.98, RMSEA = 0.028, SRMSR = 0.044) had good model fits. This indicated that the covariance matrices of the observed items were invariant across gender and grade levels. Table 5 shows that the model 0 for region comparison (χ^2^ = 1574.77, *df* = 171, CFI = 0.94, TLI = 0.89, RMSEA = 0.075, SRMSR = 0.094) had marginally acceptable model fits. This indicated that the covariance matrices of the observed items may not be invariant across regions and that further invariance tests were needed to identify the sources of invariance. No further tests of invariance for gender and grade level comparison were needed, as the covariance matrices were invariant across gender and grade levels. However, relevant tests of invariance were still tested and reported for comparison.

**Table 3:** Invariance tests across genders.

		Model Fitness							Model Comparison		
				
		χ^2^	*df*	RMSEA	CFI	TLI	SRMSR		Δ *df*	Δχ^2^	ΔCFI
**The omnibus test**											
	(0) Invariant cov. matrices	253.33	171	0.018	1.00	0.99	0.027				

**Measurement invariance tests**											
RQ1	(1) Configural invariance	1429.14	258	0.056	0.95	0.94	0.045	Baseline			
	(2) Metric invariance	1443.00	272	0.054	0.95	0.94	0.045	1 versus 2	14	13.87	0.000
	(3) Scalar invariance	1506.80	286	0.054	0.95	0.94	0.046	2 versus 3	14	63.80^∗^	-0.002

**Structural invariance tests**											
RQ2	(4) invariant factor means	1515.10	290	0.054	0.95	0.94	0.046	3 versus 4	4	8.31	0.000
RQ3	(5) invariant factor variances	1511.47	290	0.054	0.95	0.94	0.048	3 versus 5	4	4.67	0.000
	(6) invariant factor covariance	1532.41	296	0.054	0.95	0.94	0.049	5 versus 6	6	20.94^∗^	-0.001
	(7) compare structural parameters	1532.41	296	0.054	0.95	0.94	0.049	6 versus 7	0	0.00	0.000


*RQ1, research question 1; RQ2, research question 2; RQ3, research question 3; cov., covariance; ^∗^*p* < 0.01. *N* = 2913; Male *n* = 803, Female *n* = 2110; 24 participants didn’t report their gender.*

**Table 4:** Invariance tests across grade levels.

		Model Fitness							Model Comparison		
				
		χ^2^	*df*	RMSEA	CFI	TLI	SRMSR		Δ *df*	Δχ^2^	Δ CFI
**The omnibus test**											
	(0) Invariant cov. matrices	374.34	171	0.028	0.99	0.98	0.044				

**Measurement invariance tests**											
RQ1	(1) Configural invariance	1501.05	258	0.057	0.95	0.94	0.045	Baseline			
	(2) Metric invariance	1546.27	272	0.056	0.94	0.94	0.048	1 versus 2	14	45.22^∗^	-0.002
	(3) Scalar invariance	1577.39	286	0.055	0.94	0.94	0.048	2 versus 3	14	31.12^∗^	0.000

**Structural invariance tests**											
RQ2	(4) invariant factor means	1600.68	290	0.055	0.94	0.94	0.049	3 versus 4	4	23.30^∗^	-0.001
RQ3	(5) invariant factor variances	1591.81	290	0.055	0.94	0.94	0.053	3 versus 5	4	14.42^∗^	-0.001
	(6) invariant factor covariance	1601.76	296	0.055	0.94	0.94	0.055	5 versus 6	6	9.95	0.000
	(7) compare structural parameters	1601.76	296	0.055	0.94	0.94	0.055	6 versus 7	0	0.00	0.000


*RQ1, research question 1; RQ2, research question 2; RQ3, research question 3; cov., covariance. ^∗^*p* < 0.01. *N* = 2937; Primary school *n* = 1788, Secondary school *n* = 1149.*

**Table 5:** Invariance tests across mainland China (ML) and Hong Kong (HK).

		Model Fitness							Model Comparison		
				
		χ^2^	*df*	RMSEA	CFI	TLI	SRMSR		Δ *df*	Δχ^2^	Δ CFI
**The omnibus test**											
	(0) Invariant cov. matrices	1574.77	171	0.075	0.94	0.89	0.094				

**Measurement invariance tests**											
RQ1	(1) Configural invariance	1792.75	258	0.064	0.94	0.92	0.051	Baseline			
	(2) Metric invariance	1881.94	272	0.063	0.93	0.92	0.056	1 versus 2	14	89.19^∗^	-0.003
	(3) Scalar invariance	2444.35	286	0.072	0.91	0.90	0.063	2 versus 3	14	562.41^∗^	-0.023
	(3’) Partial Scalar invariance (PSI)	2037.64	281	0.065	0.93	0.92	.059	2 versus 3’	9	155.70^∗^	-0.006

**Structural invariance tests**											
RQ2	(4) Invariant factor means +(PSI)	2240.40	285	0.068	0.92	0.91	0.066	3′ versus 4	4	202.76^∗^	-0.008
RQ3	(5) Invariant factor variances +(PSI)	2054.25	285	0.065	0.93	0.92	0.064	3′ versus 5	4	16.61^∗^	-0.001
	(6) Invariant factor covariance +(PSI)	2178.79	291	0.066	0.92	0.92	0.081	5 versus 6	6	124.54^∗^	-0.005
	(7) Compare structural parameters +(PSI)	2178.79	291	0.066	0.92	0.92	0.081	6 versus 7	0	0.00	0.000


*RQ1, research question 1; RQ2, research question 2; RQ3, research question 3; cov., covariance. ^∗^*p* < 0.01. *N* = 2937; Mainland *n* = 1281, Hong Kong *n* = 1656.*

### Results of Measurement Invariance

The second sections (RQ1) of Tables 3–5 show the results for the tests of measurement invariance across genders, grade levels and regions, respectively. The results showed that the baseline model (Model 1) demonstrated good model fits for all three comparison trials. For regional comparison (Table 5), there was no significant difference between the configural invariance model (Model 1) and the metric invariance model (Model 2) (ΔCFI = -0.003 > -0.01). This indicated that the like factor loadings were invariant across the regions. However, there was a significant difference between the metric invariance model (Model 2) and the scalar invariance model (Model 3) (ΔCFI = -0.023 < -0.01). This indicated that not all like item intercepts were invariant across the regions. After loosening three invariant constraints of equal item intercepts regarding SA (Item y1, Item y2, Item y6; see Appendix) and two regarding teaching satisfaction (Item z3, Item z5; see Appendix), a partial scalar invariance model (Model 3′) was established (ΔCFI = -0.006 > -0.01) for further testing. For gender and grade level comparison, the results showed that the deceases in CFI were all less than 0.01 and there were no significant differences between Models 1 and 2 and between Models 2 and 3. The findings for gender and grade level comparison were consistent with the results of the omnibus test of invariant covariance matrices and provided justifications for adding constraints of equal factor loadings and constraints of equal item intercepts into the baseline model.

### Results of Structural Invariance

#### Invariant Factor Means

The third sections (RQ2) of Tables 3–5 show the results for the tests of invariant factor means across genders, grade levels and regions, respectively. For gender (ΔCFI = 0.000 > -0.01) and grade level comparison (ΔCFI = -0.001 > -0.01), the results showed that CFI did not decrease significantly after adding constraints of equal factor means. In other words, the factor means of SA, DA, ENFE and TS were invariant across genders and grade levels. For regional comparison (ΔCFI = -0.008 > -0.01), the results also showed that CFI did not decrease significantly after adding constraints of equal factor means to the partial scalar invariance model (Model 3′). However, the factor means of SA, DA, ENFE and TS were invariant across the regions only when five item intercepts were set free for estimation in each subgroup.

#### Invariant Variances, Covariance and Path Coefficients

The third sections (RQ3) of Tables 3–5 show the results for the tests of invariant factor variance, covariance and path coefficients across genders, grade levels and regions, respectively. Tables 3, 4 show that CFI did not decrease significantly after progressively adding constraints of equal factor variance, equal factor covariance and equal path coefficients to the scalar invariance model (Model 3) of gender comparison (ΔCFIs = 0.000, -0.001,0.000 > -0.01) and of grade level comparison (ΔCFIs = -0.001,0.000,0.000 > -0.01). In other words, the factor variance and covariance of SA, DA, ENFE and TS were invariant and the path coefficients from SA, DA and ENFE to TS were also invariant across gender and grade levels. The results in Table 5 also showed that CFI did not significantly decrease after adding these constraints of equality to the partial scalar invariance model (Model 3′) of regional comparison (ΔCFI = -0.001,-0.005,0.000 > -0.01). Similarly, the results indicated that the factor variance and covariance of SA, DA, ENFE and TS were invariant and the path coefficients from SA, DA and ENFE to TS were also invariant across the regions when five item intercepts were set free for estimation in each subgroup.

### Results of Structural Equation Modeling

**FIGURE 1:**
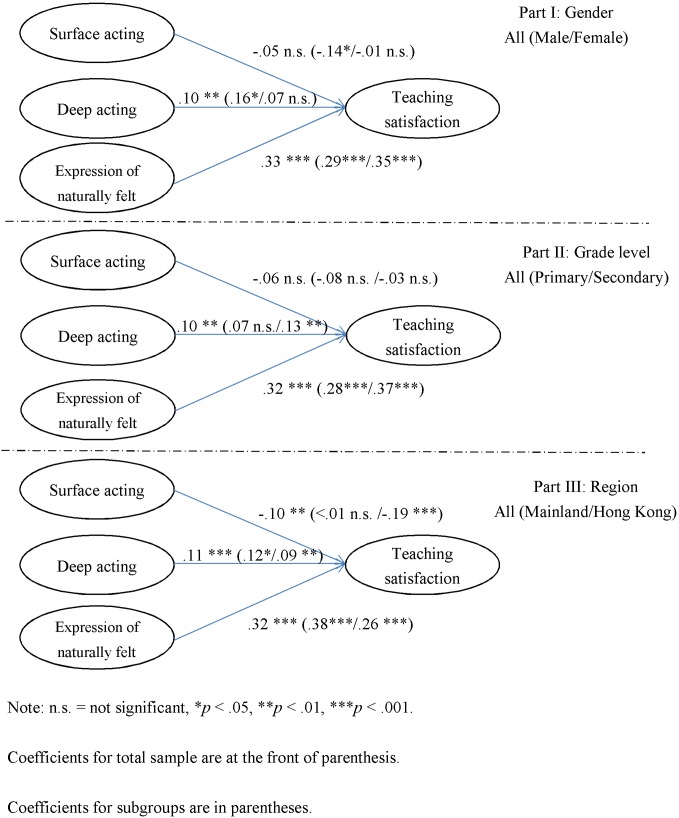
The results of structural equation modeling.

Figure 1 shows the results of the SEM. Both path coefficients for the total sample (with constraints of equal path coefficients) and those for subgroups (with no constraints of equal path coefficients) are presented. Overall, DA and ENFE were positively and significantly related to teaching satisfaction, and the relationships between DA and teaching satisfaction was weaker than those between ENFE and teaching satisfaction. Thus, H1b and H1c were supported. Nevertheless, SA was found to be negatively or not related to teaching satisfaction. That is, the relationships between SA and teaching satisfaction were weaker than expected, and H1a was not supported. Moreover, while the tests of statistical significance indicated no significant difference between models with and without constraints of equal path coefficients, the comparisons between the subgroup coefficients revealed some interesting findings. For gender comparison, SA was negatively related to (β = -0.14, *p* < 0.05), while DA was positively related to (β = 0.16, *p* < 0.05), males’ teaching satisfaction. There were no significant relationships found between females’ teaching satisfaction and their use of surface (β = -0.01, n.s.) or DA (β = 0.07, n.s.). For grade level comparison, DA was significantly and positively related to secondary school teachers’ teaching satisfaction (β = 0.13, *p* < 0.01), but not for primary school teachers (β = 0.07, n.s.). Similarly, for regional comparison, SA was significantly and negatively related to Hong Kong teachers’ teaching satisfaction (β = -0.19, *p* < 0.001), but not to teachers from mainland China (β < 0.01, n.s.). In other words, when it comes to H2a, H2b, and H2c, it was found that there was no difference of ‘kind’ but some differences of ‘degree’ observed across genders, grade levels and regions.

## Discussion

While studies have generally suggested that males and females, primary and secondary school teachers, and people from different regions/cultures may perceive and regulate their emotions differently, no research has examined the measurement invariance in terms of teachers’ emotional labor and its effects on their well-being. This study took a step forward by investigating this issue in Hong Kong and mainland China. Hong Kong is perceived as a bridge between Chinese and Western societies (Li and Bray, 2007). The comparisons between the two regions with both cultural and social diversities and similarities are meaningful and contribute to the knowledge of detailed regional differences within China (Wong et al., 2007; Fung and You, 2011). The results of this study, in general, indicated no significant individual differences. This suggested that there was no difference of ‘kind’ between teachers from Hong Kong and mainland China. Meanwhile, this study revealed that some differences of ‘degree’ exist between the two samples, and are worthy of further discussion.

The results of this study showed gender, grade level and cultural/regional invariance of the ELSs measurements. Based on the suggestions of Vandenberg and Lance (2000), a series of tests of measurement invariance were conducted and the results indicated that the three-factor measurement of ELSs was generally robust and invariant, and could be used to measure the emotional labor of different teacher groups. The data were thus reliable and could be used to test further substantive hypotheses and provide meaningful interpretations. However, only a partial scalar invariance model has been established for the regional comparison. The results indicated that there were systematic differences in responses to the offending items (Items y1, y2, y6; Items z3, z5; see Appendix). In other words, teachers from Hong Kong and mainland China responded differently to these items even after controlling for the latent factors. Thus, traditional statistics that rely on item mean score comparisons (e.g., *t*-tests) would be problematic (Fischer, 2004). Further, Vandenberg and Lance (2000) suggested that the violation of scalar invariance may be due to undesirable rating biases or true group differences. The offending items of SA contained expressions of putting on ‘an act,’ ‘a show’ and ‘a mask,’ and the differences between teachers from Hong Kong and mainland China may be due to social desirability bias or because of sub-cultural differences in the emotional coloring of the words. Qualitative studies on these issues may provide further detail.

The results also indicated that these categorical factors (i.e., genders, grade levels, and regions) did not moderate the relationships between ELSs and teaching satisfaction. In general, SA was not related or negatively related to teaching satisfaction. DA and ENFE were positively related to teaching satisfaction. While SA was previously found to be a most maladaptive way of emotion regulation, the relationships between SA and teaching satisfaction found in this present study were quite weak. A possible explanation for these unexpected results may be that SA was more closely related to affective outcomes such as emotional exhaustion than cognitive outcomes such as teaching satisfaction (Yin et al., unpublished). Teachers’ job satisfaction is a comprehensive construct that should be considered beyond the affective aspects but to wider occupational contexts (Kinman et al., 2011). The results also suggested that emotional labor was less stressful or deleterious for teachers than other service employees (Humphrey et al., 2015; Yin et al., 2017).

Furthermore, although not statistically significant, the effects of SA and DA on teaching satisfaction were more salient in male teachers, secondary school teachers, and teachers from Hong Kong. Previous literatures suggested that emotional suppression was quite common among females who were generally in a low social position and teachers from mainland China, who generally accept high power distances (Hargreaves, 2000; Fung and You, 2011). As a result, their SA was relatively tolerable and did not necessarily lead to job dissatisfaction. For primary school teachers who have more authority to express intensive emotions, both positive and negative, in the classroom (Hargreaves, 2000), both SA and its negative effects were minimal. These results are consistent with previous claims that emotional labor is less stressful or has less influence in collectivist cultures that advocate social harmony and interdependence among people (Allen et al., 2014; Mastracci and Adams, 2018). In other words, personal identity and social identity will influence how individuals evaluate the situations, and further moderate the relationships between ELS and its outcomes, especially when it comes to the cognitive outcomes such as teaching satisfaction (Shamir et al., 1993; Humphrey et al., 2015).

The lack of statistical significance in terms of the moderating effects of gender/grade level/region may indicate that although slight differences could be observed between males and females, primary and secondary school teachers, and Hong Kong and mainland Chinese teachers, these results should not be taken as evidence for some stable and stereotypical images of the subgroups. Due to the processes of internalization and globalization, the differences between collectivistic and individualistic approaches become blurred, particularly among the younger generations (Fung and You, 2011). Researchers have also suggested that personal experiences may override the effects of cultural imperatives and affect individuals’ behavioral patterns and emotional functioning (Kennedy et al., 2004). In primary schools, female teachers, similar to their male counterparts, have substantive power and tend to express their emotions freely and directly (Hargreaves, 2000).

This study had three key limitations. First, the evidence of measurement and structural invariance was based on two sets of data with large sample sizes. The two samples involved in the study were not representative of groups in Hong Kong or mainland China. The generalisability of our findings was thus limited. More studies are consequently needed to obtain representative samples and replicate the results. Second, for the indicator of teacher well-being, this study only concerned teachers’ job satisfaction. When different indicators of teacher well-being (e.g., emotional exhaustion) are adopted, the paths between emotional labor and well-being indicators could be different. Hence, future studies are suggested to use some more comprehensive assessments of teacher well-being. Third, because of the cross-sectional design of the studies, this study could not demonstrate any causal relationship. Longitudinal research designs or experimental studies are thus needed to obtain stronger evidence.

## Conclusion

Numerous studies have focused on emotional labor and differences in terms of gender, age, grade level, and culture among individuals in general and teachers in particular. However, few of these studies have used quantitative methods and been based on the prerequisites of measurement invariance (Hargreaves, 2000; Marston et al., 2005; Johnson and Spector, 2007). By using multi-group SEM, this study has contributed to the understanding of individual differences in terms of teachers’ emotional labor and its relationship with teaching satisfaction. We also contribute to the issue of methodological rigor by testing both measurement and structural invariance models across gender, grade levels and regions (Hong Kong and mainland China). While differences have been observed across genders, grade levels and regions, the significance was found to be minimal. The implications of the present study are threefold. First, our results provide preliminary support for the generalisability of the three-dimensional emotional labor strategies scale in teacher samples. Nevertheless, researchers need to be careful when using traditional statistics. Second, our results generally support the robust relationships between emotional labor strategies and teaching satisfaction but highlight the necessity of distinguishing between affective and cognitive outcomes. Third, the results show no difference of ‘kind’ but some differences of ‘degree’ observed across genders, grade levels and regions. Individuals’ authority in the workplace and their acceptance of power distance are important factors influencing their use of ELSs and appraisals of theirs jobs.

## Author Contributions

SH analyzed the data and wrote the first version of the manuscript. HY designed the research and finished the final version of the manuscript. JH collected the data and helped with the data analysis.

## APPENDIX

Surface acting

y1. I put on an act in order to deal with students and parents in an appropriate way.

y2. I put on a “show” or “performance” when interacting with students and parents.

y3. I show feelings to students and parents that are different from what I feel inside.

y4. I fake the emotions I show when dealing with students and parents.

y5. I just pretend to have the emotions I need to display for my job.

y6. I put on a “mask” in order to display the emotions I need for the job.

Deep acting

y7. I try to actually experience the emotions that I must show to students and parents.

y8. I make an effort to actually feel the emotions that I need to display toward students and parents.

y9. I work hard to feel the emotions that I need to show to students and parents.

y10. I work at developing the feelings inside of me that I need to show to students and parents.

Expression of naturally felt emotions

y11. The emotions I express to students and parents are genuine.

y12. The emotions I show students and parents come naturally.

y13. The emotions I show customers match what I spontaneously feel.

Teaching satisfaction

z1. In most ways, being a teacher is close to my ideal.

z2. My conditions of being a teacher are excellent.

z3. I am satisfied with being a teacher.

z4. So far I have gotten the important things I want to be a teacher.

z5. If I could choose my career over, I would change almost nothing.
